# 

**Published:** 1877-08-20

**Authors:** 


					﻿VOL. Vn.	AUGUST 20, 1877	No. 8.
THE
SOOTHERS MEDICAL RECORD.
A MONTHLY JOURNAL OF PRACTICAL MEDICINE.
QUICQUID PR2ECIPIES ESTO BIIEVIS.”
K
W
Ph
M
W
Ph
i-q
◄
O
i—i
Q
W
a*
A
'!□
o
>*
o
H
i-q
<1
P5
o
CC
i—i
M
Ph
O
QQ
S
o
H
Pl
Eh
W
QQ.
H
Ph
Ph
H
QQ
w
i-q
On
QQ
M
o
SJ
Q
O
S
3
g
a
►
i—i
O
3
QQ
>
d
SI
>
3
£H
H
QQ
QQ
O
d
£<
H
H
d
JAS. P. HARRISON & CO., Printers.
ATLANTA, GEORGIA.
TERMS: $2.00 per Annum in Advance—Club Rates, 5 Copies for $8.00.
				

